# Nasal Delivery of Hesperidin/Chitosan Nanoparticles Suppresses Cytokine Storm Syndrome in a Mouse Model of Acute Lung Injury

**DOI:** 10.3389/fphar.2020.592238

**Published:** 2021-01-27

**Authors:** Hua Jin, Zuguo Zhao, Qian Lan, Haotong Zhou, Zesen Mai, Yuan Wang, Xiaowen Ding, Wenting Zhang, Jiang Pi, Colin E. Evans, Xinguang Liu

**Affiliations:** ^1^ College of Pharmacy, Guangdong Provincial Key Laboratory of Medical Molecular Diagnostics, Guangdong Medical University, Dongguan, China; ^2^ Department of Microbiology and Immunology, University of Illinois at Chicago, Chicago, IL, United States; ^3^ Feinberg School of Medicine, Northwestern University, Chicago, IL, United States

**Keywords:** cytokine storm syndrome, hesperidin, chitosan nanoparticle, lung inflammation, nasal drug delivery

## Abstract

The cytokine storm or cytokine storm syndrome (CSS) is associated with high mortality in patients with acute lung injury (ALI) and acute respiratory distress syndrome (ARDS), for example following sepsis or infectious diseases including COVID-19. However, there are no effective treatments for CSS-associated ALI or ALI/ARDS. Thus, there remains an urgent need to develop effective drugs and therapeutic strategies against CSS and ALI/ARDS. Nasal and inhaled drug delivery methods represent a promising strategy in the treatment of inflammatory lung disease as a result of their ability to improve drug delivery to lungs. Improving the nasal mucosa absorption of poorly water-soluble drugs with poor mucosa bioavailability to a therapeutically effective level is another promising strategy in the fight against ALI/ARDS. Here, chitosan nanoparticles loaded with hesperidin (HPD/NPs) were developed for nasal delivery of the anti-inflammatory HPD compound to inflammatory lungs. *In vitro* and *in vivo*, HPD/NPs exhibited enhanced cellular uptake in the inflammatory microenvironment compared with free HPD. In a mouse model of inflammatory lung disease, the HPD/NPs markedly inhibited lung injury as evidenced by reduced inflammatory cytokine levels and suppressed vascular permeability compared with free HPD. Collectively, our study demonstrates that nasal delivery of HPD/NPs suppresses CSS and ALI/ARDS in a murine model of inflammatory lung disease, and that nanoparticle-based treatment strategies with anti-inflammatory effects could be used to reduce CSS and ALI in patients with inflammatory lung injury.

## Introduction

Inflammatory lung injury, including sepsis-induced acute lung injury (ALI) and acute respiratory distress syndrome (ARDS), is associated with increased expression of pro-inflammatory cytokines. This so-called cytokine storm syndrome (CSS) is characterized by excessive amounts of pro-inflammatory cytokines, such as the interleukins (IL), interferons (IFN), and tumor necrosis factors (TNF) (Gupta et al., 2020). CSS is associated with clinical deterioration and high mortality in patients with viral infections, such as COVID-19 (Mehta et al., 2020), SARS (Channappanavar and Perlman, 2017) and influenza. At present, there are no efficient drugs or strategies to treat CSS and CSS-associated ALI/ARDS in severe COVID-19 cases.

Although corticosteroids have been used to inhibit CSS in COVID-19 patients, such drugs are limited by their side effects (Russell et al., 2020). Furthermore, several antiinflammatory agents have been shown to reduce ALI and inflammation in experimental studies, but have failed in clinical trials of ALI/ARDS patients (Matthay et al., 2017). Alternative anti-inflammatory methods targeting CSS are under development, such as interleukin-6 antibodies, and have been used to combat COVID-19 (Zhang et al., 2020). Long-term use of antibody treatments, however, could cause chronic immunosuppression. Thus, the development of novel therapies to effectively control and target ALI and CSS is an urgent requirement in the fight against sepsis-induced ALI/ARDS, COVID-19, or other infectious inflammatory lung diseases. One potential limitation of anti-inflammatory agents is the off-target delivery to, and short-term retention of, the agents in lungs. In this work, a nasal nanoparticle (NP)-based drug delivery system was designed to deliver an inflammatory agent (Hesperidin, HPD) to inflammatory lungs with the view to reducing CSS and ALI/ARDS. HPD is an active flavonoid aglycone (Hemanth Kumar et al., 2017) found in citrus fruits, which has shown no side effects or toxicity in experimental animal studies (Li et al., 2019). Along with its anti-inflammatory properties, HPD exhibits analgesic, anti-carcinogenic, anti-viral (Ciftci et al., 2015), and anticoagulant activities (Roohbakhsh et al., 2015).

Many investigators are aiming to improve the development of drug-loaded NPs and the delivery of drugs to infectious sites via cell- or tissue-specific targeting techniques (Wang et al., 2018). Nasal drug delivery systems can be employed to reduce elimination by the liver, kidneys, and gastrointestinal tract (Sukumar et al., 2019), compared with oral, intraperitoneal, or intravenous delivery routes. However, nasal administration of HPD is limited *in vivo* because of its poor aqueous solubility and bioavailability. To address this drawback, PLGA or chitosan nanoparticle (NP) delivery systems have been developed to improve the aqueous solubility, safety, and efficacy of pharmacological agents (Jin et al., 2017). Chitosan (CS)-based NPs, for example, can significantly improve mucosal drug delivery, as a result of the electrostatic attraction between positively charged CS chains and negatively charged sialic acid of nasal mucosa (Gholizadeh et al., 2019).

In this work, we synthesized an HPD-loaded PLGA-CS NP delivery system (HPD/NPs) to target HPD to inflammatory lungs via the nasal delivery route, and assessed the capacity of these HPD/NPs to suppress CSS and ALI in a mouse model of endotoxic lung injury.

## Methods and Materials

### Preparation and Characterization of Hesperidin-Loaded PLGA-Chitosan NPs (HPD/NPs)

HPD-NPs were synthesized by emulsification and evaporation methods. Briefly, 20 mg HPD and 80 mg PLGA-PEG (1:1 lactide: glycolide, Sigma, USA) were dissolved in 5 ml of dichloromethane and homogenized for 40 seconds to form the oil phase emulsification. The oil phase emulsification was combined with 20 ml PVA (1% w/w, Sigma, USA) containing of 0.2% of chitosan and homogenized for another 40 s to form the second water phase emulsification. This water phase emulsification was added to 100 ml water and stirred for 6 h to achieve organic reagent evaporation and nanoparticle hardening. Finally, HPD/NPs were harvested by centrifugation at 12,000 rpm for 20 min and washed 3 times with ultrapure water then lyophilized for 48 h for storage at 4 °C.

### Characterization of NPs

Size distribution and zeta potential were estimated by dynamic laser scattering using a Zetasizer Nano ZS (Malvern Instruments, UK). NPs were visualized by scanning electron microscopy (Philips Co, Holland). For measuring HPD loading capacity in HPD/NPs, 10 mg lyophilized NPs were dissolved in 1 ml of dichloromethane, and then, the amount of HPD in the solution was determined by high-pressure liquid chromatography (HPLC). HPLC detection was performed using a C18 column (5 μm, 250 mm × 4.6 mm), whereas the mobile phase, consisting of methanol and 0.1% acetic acid (88:12) (v/v), was maintained at a flow rate of 1.0 ml/min. The ultraviolet detector wavelength was 285 nm, and the injection volume was 20 μl.

### Evaluation of HPD Release *in vitro*

HPD release from NPs was evaluated *in vitro* using the dialysis method as previously described (Jin et al., 2017). Briefly, dialysis bags with a molecular weight cut-off of 10,000 Da containing 10 mg of compounds were immersed in a water bath containing 20 ml of PBS (pH 7.4) at 37 °C. At indicated times, 1 ml of receiving buffer was withdrawn and replaced with 1 ml of PBS. HPD release from dialysis bag into the water bath was determined by ultraviolet spectrophotometry (Agilent 8,453, Agilent Technologies, USA) at 285 nm.

### Cell Culture

Mouse macrophage RAW264.7 cells and human umbilical vein endothelial cells (HUVECs, Lonza) were cultured in DMEM (Gibco) with 10% fetal bovine serum (Gibco) containing 100 µg/ml streptomycin and 100 IU/ml penicillin. Cells were sub-cultured twice/week and incubated in a humidified incubator (Thermo) at 5% CO2 and 37 °C.

### Cell Viability Assay

RAW264.7 cells or HUVECs were cultured in 96-well plates for 24 h, then exposed to 10 µg/ml HPD or HPD/NPs or vehicle (0.02% DMSO) for 3 h, followed by activation with 1 µg/ml of LPS for 24 h. Cell viability was assessed using the Cell Counting Kit assay (Beyotime Institute of Biotechnology, Nanjing, China) according to the manufacturer’s instructions. All experiments were performed three times.

### Endothelial Permeability Assay

The endothelial permeability assay was performed as described (Monfoulet et al., 2017) with the following modifications. In brief, HUVECs were seeded onto Corning Transwell filters for 24 h in medium with 0.2% FBS then exposed to 10 µg/ml HPD or HPD/NPs or vehicle (0.02% DMSO) for 3 h, followed by activation with 1 µg/ml of LPS for 24 h. Permeability of the endothelium was evaluated by assessing the passage of FITC-dextran (40 kDa) through endothelial monolayer. One hundred microliters of FITC-dextran were added to the upper chamber and allowed to equilibrate for 1 h, after which FITC fluorescence (excitation 488 nm; emission 525 nm) in the lower chamber was measured using a microplate reader. Three independent experiments were performed.

### Immunofluorescence Staining

F-actin expression was evaluated by staining with phalloidin FITC. In brief, HUVECs were cultured on coverslips, then fixed with 4% paraformaldehyde for 30 min and incubated with 1 mM phalloidin-FITC for 60 min in the dark at room temperature, and then washed twice with PBS. Cytoskeleton organization was imaged with a laser scanning confocal microscope (LCM 880, Carl Zeiss, Germany). Fluorescence was measured by flow cytometer at excitation wavelength 488 nm, emission wavelength 530 nm to quantitatively elucidate the alterations of cytoskeleton proteins.

HUVECs cultured on coverslips were fixed with 4% paraformaldehyde and stained with primary anti VE-cadherin (1:1,000; Sigma-Aldrich) and fluorescence-conjugated secondary antibodies (1:500; Sigma-Aldrich). Nuclei were counterstained with DAPI (DAPI Fluoromount-GTM, thermofisher). Cells were imaged with LCM 880.

### *In vivo* Studies

#### Mice

C57BL/6 mice were used throughout at 12–14 weeks of age. The experimental protocols were conducted according to National Institutes of Health guidelines on the use of laboratory animals. The animal care and study protocols were approved by the Institutional Animal Care and Use Committee of Guangdong Medical University (GDY2002094).

#### Mouse Model of Acute Lung Injury

Lipopolysaccharide (LPS, *E. coli* 055:B5, Santa Cruz), a component of the cell wall of Gram-negative bacterium, was dissolved in PBS. To induce sepsis, LPS was administered intraperitoneally to mice at 3.5 mg/kg body weight in 100 µL PBS. HPD or HPD/NPs was nasally administrated at 3 h-post LPS challenge, and the animals were sacrificed to collect samples at 24 h post-LPS challenge.

#### Histology

Lung tissues were fixed and processed for H&E staining. Briefly, lung tissues were fixed by 5 min instillation of 10% PBS-buffered formalin through trachea catheterization at a transpulmonary pressure of 15 cm H_2_O, and then overnight at 4°C with agitation. After paraffin processing, the tissues were cut into 5 µm sections and stained with H&E for histological analysis.

#### Assessment of Lung Vascular Permeability

The Evans blue-conjugated albumin (EBA) extravasation assay was used to assess pulmonary vascular permeability (Huang et al., 2016). Briefly, EBA (20 mg/kg) was injected retro-orbitally at 45 min before sacrifice and lung collection following perfusion free of blood with PBS. The extravasated EBA in lung homogenates was expressed as μg of EBA per g of lung.

Total protein levels in bronchiolar alveolar lavage fluid (BALF) were measured via bicinchoninacid-assay (BCA) according to the manufacturer’s instructions (Pierce BCA Protein Assay, Thermo Scientific, USA).

#### Immunohistochemistry

The Lung sections were incubated overnight at 4 °C in a humidified chamber with anti-caspase 1 (Proteintech: 22915-1-AP) or anti- IL-1β antibodies (Abcam: ab9722) diluted 1:500 in PBS containing 1% BSA. Primary antibodies were incubated at 4 °C overnight followed by secondary antibodies for 1 h at 37 °C. Proteins were visualized using the DAB chromogen kit (ZSGB-BIO, Beijing, China). Finally, the lung sections were counter-stained with hematoxylin.

### Statistical Analysis

Results are expressed as mean ± SD. Statistical significance was determined by 1-way ANOVA with a Games-Howell post hoc analysis for multiple-group comparisons. Two-group comparisons were analyzed by the 2-tailed unpaired Student *t*-test.

## Results

### Preparation and Characterization of HPD/NPs

**FIGURE 1:**
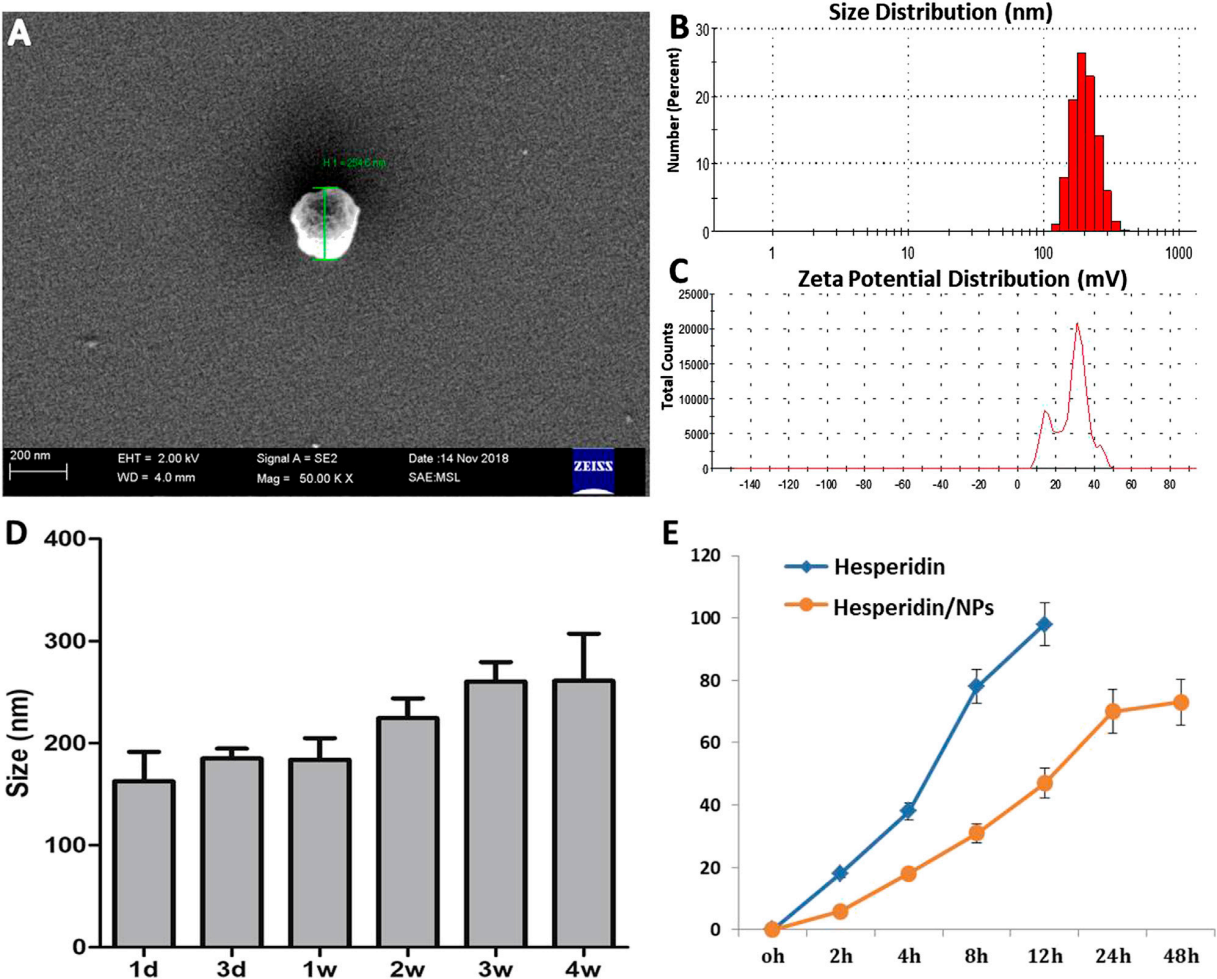
Characterization of HPD/NPs. **(A)** Scanning electron microscopy (SEM) image of an HPD/NP in suspension. **(B)** Mean size and **(C)** zeta potentials of HPD/NPs. **(D)** HPD/NP size over time (*n* = 4/group). **(E)** *In vitro* release of HPD from HPD/NPs in PBS (0.01 M, PH = 7.4, *n* = 4/group).

To improve the water solubility and bioavailability of HPD, biodegradable polymer PLGA was employed to encapsulate HPD to form soluble NP carriers, and chitosan was employed to modulate the surface zeta potential of the NPs to positive (Bruinsmann et al., 2019). After formulation of the HPD/NPs, the particle size, zetapotential, morphology, entrapment efficiency, and HPD release were determined by dynamic laser light scattering (DLS), scanning electronic microscopy (SEM) and HPLC, respectively. The size distribution of HPD/NPs was approximately 200 nm (Figures 1A,B). The surface charge (zeta potential) was +22 mV (Figure 1C). The loading rate of HPD into the NPs was 8.1%, and encapsulation rate was 81.02%. The HPD/NP diameter did not change significantly for up to 4 weeks at 37 °C (Figure 1D). The release of HPD from the HPD/NPs occurred steadily in the first 12 h, with ∼75% and ∼90% of the HPD being released by 12 and 24 h, respectively (Figure 1E). Notably, free NPs had a similar appearance and diameter to HPD/NPs (Supplementary Figure S1A).

### *In vitro* Anti-Cytokine Storm Syndrome Activity of HPD and HPD/NPs

**FIGURE 2:**
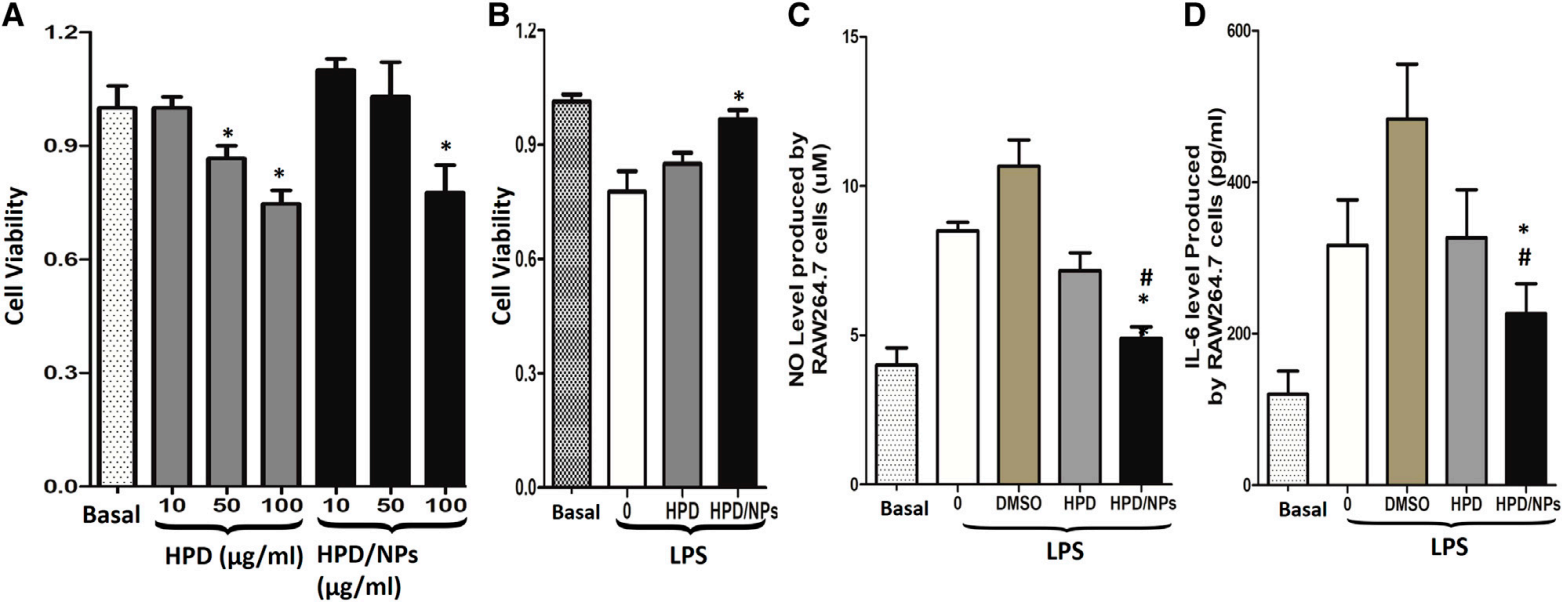
Impact of HPD and HPD/NPs on LPS-induced inflammatory response *in vitro*. **(A)**. Viability of RAW264.7 macrophages exposed to different doses of HPD or HPD/NPs for 24 h. **(B)** Viability of RAW264.7 macrophages exposed to 10 mg/ml HPD or HPD/NPs or vehicle (0.02% DMSO) for 3 h, followed by stimulation with 1 µg/ml of LPS for 24 h. **(C)** NO and **(D)** IL-6 production by RAW264.7 macrophages exposed to 10 mg/ml HPD or HPD/NPs or vehicle (0.02% DMSO) for 3 h, followed by stimulation with 1 µg/ml of LPS for 24 h **(A)** *N* = 4/group, *p < 0.05 vs. basal group or 10 μg/ml group. **(B–D)**. *N* = 4/group, *p < 0.05 vs. vehicle control; #*p* < 0.05 vs. HPD group.

Figure 2A showed that 10 or 50 µg/ml of HPD or HPD/NPs did not induce toxic effects on RAW264.7 cells, while 100 µg/ml of HPD or HPD/NPs caused decreases in cell viability. Therefore, 10 µg/ml concentrations of HPD or HPD/NPs were selected as the dose in the following *in vitro* and *in vivo* experiments. Next, RAW264.7 cells were treated with LPS (1 µg/ml) for 24 h. Figure 2B showed that the decreases in cell viability resulting from LPS exposure could be restored by pre-treatment with 10 µg/ml of HPD/NPs, demonstrating that HPD/NPs can protect against LPS-induced cell death *in vitro*. Figures 2C,D indicate that 10 µg/ml of HPD/NPs can significantly suppress the level of inflammation cytokines (NO and IL-6) in LPS-treated RAW264.7 cells. Notably, 2% DMSO aggravated the inflammatory profile of the RAW264.7 cells, which implied that the DMSO-free delivery of water-soluble HPD/NPs could decrease the side effects of DMSO-based drug delivery. We also found that free NPs did not alter LPS-induced cell viability vs. vehicle-treated cells (Supplementary Figure S1B).

### HPD/NPs Preserved Endothelial Permeability *in vitro*

**FIGURE 3:**
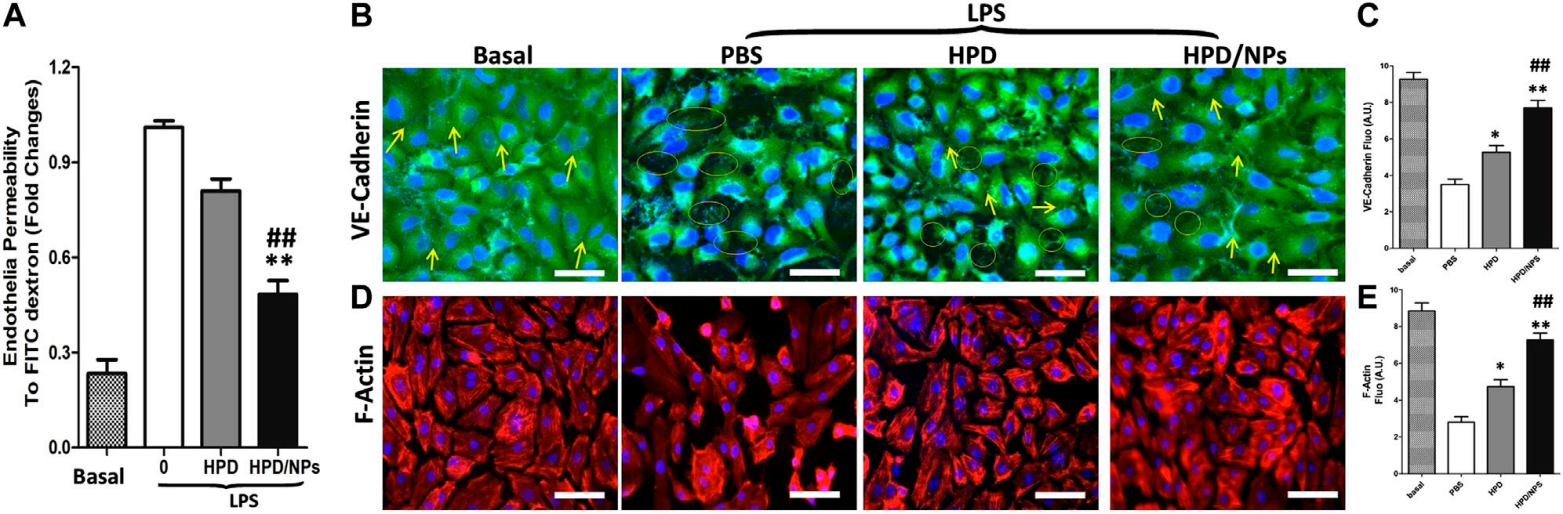
Impact of HPD and HPD/NPs on LPS-induced endothelial integrity *in vitro*. **(A)** Permeability of the HUVEC monolayer to FITC-dextran (40 kDa) after 3 h exposure to PBS or HPD or HPD/NPs (10 µg/ml) followed by 24 h LPS exposure. Data expressed as a percentage of PBS + LPS treatment group (*n* = 4/group, **p < 0.001 vs. vehicle control group; ##*p* < 0.001 vs. HPD group). **(B)** Representative images and **(C)** quantification of immunostaining for VE-cadherin (green) in HUVEC monolayers Arrows indicate VE-cadherin-positive cell junctions; circles indicate weak or absent VE-cadherin signal at cell junction. **(D)** Representative images and **(E)** quantification of F-actin (red) immunostaining of HUVECs after 3 h exposure to PBS or HPD or HPD/NPs followed by 24 h LPS (3.5 mg/kg). Nuclei were counterstained with DAPI (blue). Scale bar, 50 μm *p < 0.05 and **p < 0.01 vs. PBS group; ##*p* < 0.01 vs. HPD group. A.U., arbitrary units.

To delineate the effects of HPD and HPD/NPs on regulating endothelial barrier function, we employed a Transwell system to quantify changes in the integrity of endothelial junctions activated by LPS. HUVECs were plated at >90% confluence on Transwell filters to form cell–to-cell contacts and intact monolayers before treatment with compounds and/or LPS. Upon addition of PBS, HPD or HPD/NPs for 2 h, followed by activation with LPS for 24 h, the PBS-treated group exhibited significantly increased permeability compared with the LPS-free basal group, indicative of decreased endothelial barrier function. Figure 3A shows that stimulation of HUVECs by LPS resulted in an approximately 5-fold increase in endothelial permeability compared to the basal group. This increase in permeability was inhibited by 25% and 58% following pre-incubation with 10 µg/ml of HPD and HPD/NPs, respectively. Interestingly, there was no significant change in endothelial permeability in the cells treated with 10 µg/ml HPD/NPs compared with the basal group, indicative of restored endothelial barrier function. To assess the morphological properties of the HUVEC monolayers, VE-cadherin and F-actin expression was observed by immunofluorescence microscopy. Figure 3B,E shows that LPS induced EC shrinkage and decreased expression of VE-cadherin and F-actin. Excitingly, 10 µg/ml of HPD/NPs could restore normal EC shape and reverse the reduced expression of VE-cadherin and F-actin. Treatment with 10 µg/ml of free HPD, however, failed to decrease the LPS-induced increases in HUVEC permeability or restore VE-cadherin and F-actin expression. These studies showed that HPD/NPs can be used to alleviate LPS-induced increases in endothelial permeability and morphology disruption.

### *In vivo* Inhibition of Lipopolysaccharide-Induced Cytokine Storm by HPD/NPs

**FIGURE 4:**
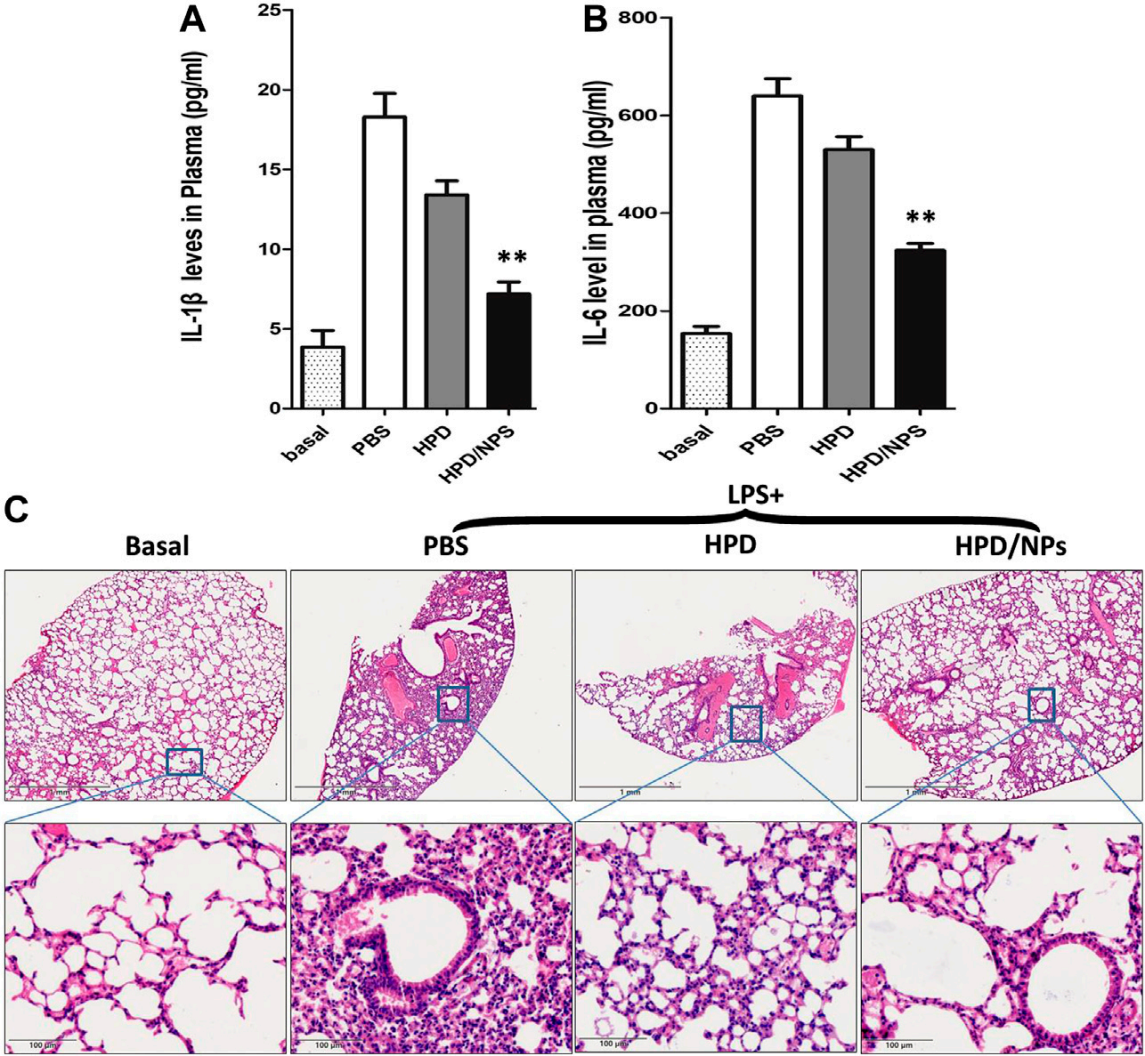
Impact of HPD and HPD/NPs on LPS-induced inflammation in mice. At 3 h postLPS, PBS (vehicle), HPD, or HPD/NPs were nasally administered to mice. Lung tissues were collected at 24 h post-LPS challenge. **(A)** Expression levels of IL-1β and **(B)** IL-6 in mouse plasma at 24 h post-LPS challenge (*n* = 4/group; **p < 0.001 vs. PBS vehicle and vs. HPD). **(C)** Representative micrographs of H&E stained lung tissue cross-sections at 24 h post-LPS challenge. Scale bar, 1 mm (upper row) or 100 μm (lower row).

The effects of HPD or HPD/NPs were further examined *in vivo* in a mouse sepsis model. LPS injection is commonly used to induce ALI (Lei et al., 2018) and CSS (Du et al., 2018). First, we confirmed that treatment of LPS mice with blank NPs did not alter the level of lung injury vs. LPS mice, as shown by absence of change in BALF protein concentration (Supplementary Figure S1C). Figures 4A,B demonstrates that IL-1β and IL-6 levels in peripheral blood promptly ascended at 24 h after intraperitoneal injection of LPS.

Treatment with 10 mg/kg of HPD/NPs efficiently depressed the LPS-induced increases in these inflammatory factors over this short period of time. Moreover, pro-inflammation cytokines such as TNF-α and IL-17 play crucial roles in lung inflammation, so we also determined the levels of such cytokines in BALF. As shown in Supplementary Figure S2A, B, treatment with HPD/NPs inhibited the LPS-induced increases in these inflammatory cytokines. In addition to pro-inflammatory cytokines, the LPS-induced expression of NO (Supplementary Figure S2C) was also decreased by HPD/NPs treatment. Treatment with blank NPs, however, had no discernable effects on inflammatory cytokine levels or NO production in LPS-treated mice, which implied that HPD acts as the anti-inflammatory agent, while the chitosan NPs act as passive drug carriers. Consistent with these findings, H&E staining showed inflammatory cell infiltration in the lungs of LPS-treated mice (Figure 4C), while treatment with HPD/NPs effectively alleviated the infiltration of inflammatory cells.

### HPD/NPs Reduce Lung Injury and Vascular Permeability

**FIGURE 5:**
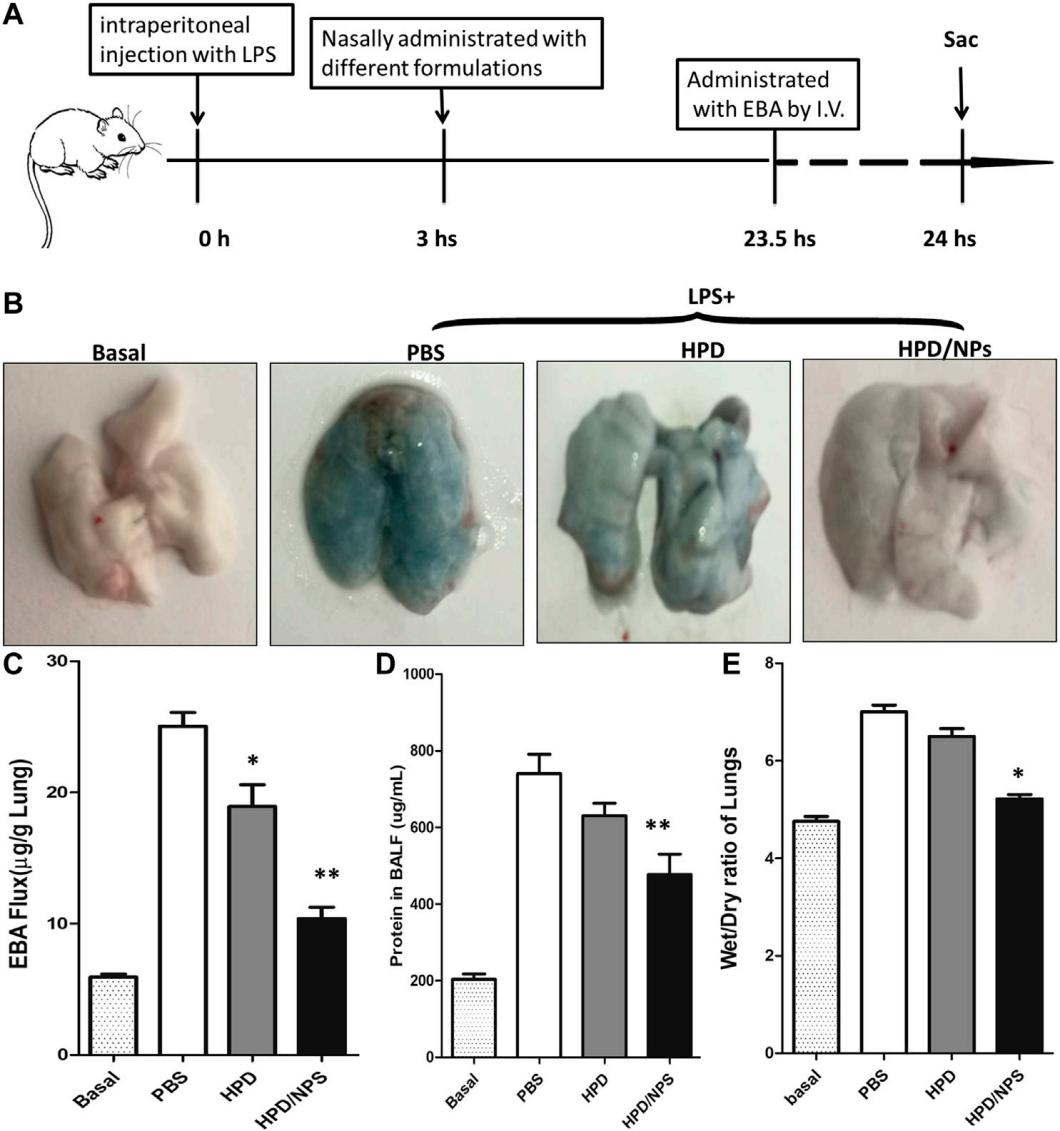
Impact of HPD and HPD/NPs on LPS-induced ALI in mice. At 3 h post-LPS, PBS (vehicle), HPD, or HPD/NPs were nasally administered to mice. Lung tissues were collected at 24 h post-LPS challenge. **(A)** EBA assay schematic. **(B)** Representative images of murine lung tissues after EBA-injection. **(C)** EBA flux, **(D)** BAL protein, and **(E)** wet/dry weight ratio. **(C–E)** N = 5; *p < 0.05 and **p < 0.001 vs. PBS group.

We next investigated the impact of the HPD/NPs on LPS-induced lung injury. We determined alterations in vascular permeability by assessing pulmonary transvascular flux of Evans blue dye-conjugated albumin (EBA) (Huang et al., 2016), bronchiolar-alveolar lavage (BAL) protein and wet/dry ratio of lungs. Figure 5A demonstrates the experimental scheme of the EBA assay. Figure 5B shows representative images of lungs extracted from EBA-injected-mice. As expected, LPS treatment resulted in increases in EBA flux, BAL protein, and wet/dry ratio at 24 h post-LPS compared to basal controls (Figures 5C–E). In mice receiving HPD at 3 h post-LPS, there were no discernable improvements in wet/dry ratio or BAL protein at 24 h post-LPS compared to PBS treatment. Mice receiving HPD/NPs, however, showed decreased levels of LPS-induced vascular permeability (EBA flux and BAL protein), and edema (wet/dry ratio). These data demonstrated that nasally administered HPD/NPs result in targeted delivery of HPD to the inflammatory lungs and inhibition of lung injury.

### HPD/NPs Suppress Pyroptosis in Septic Mouse Lungs

**FIGURE 6:**
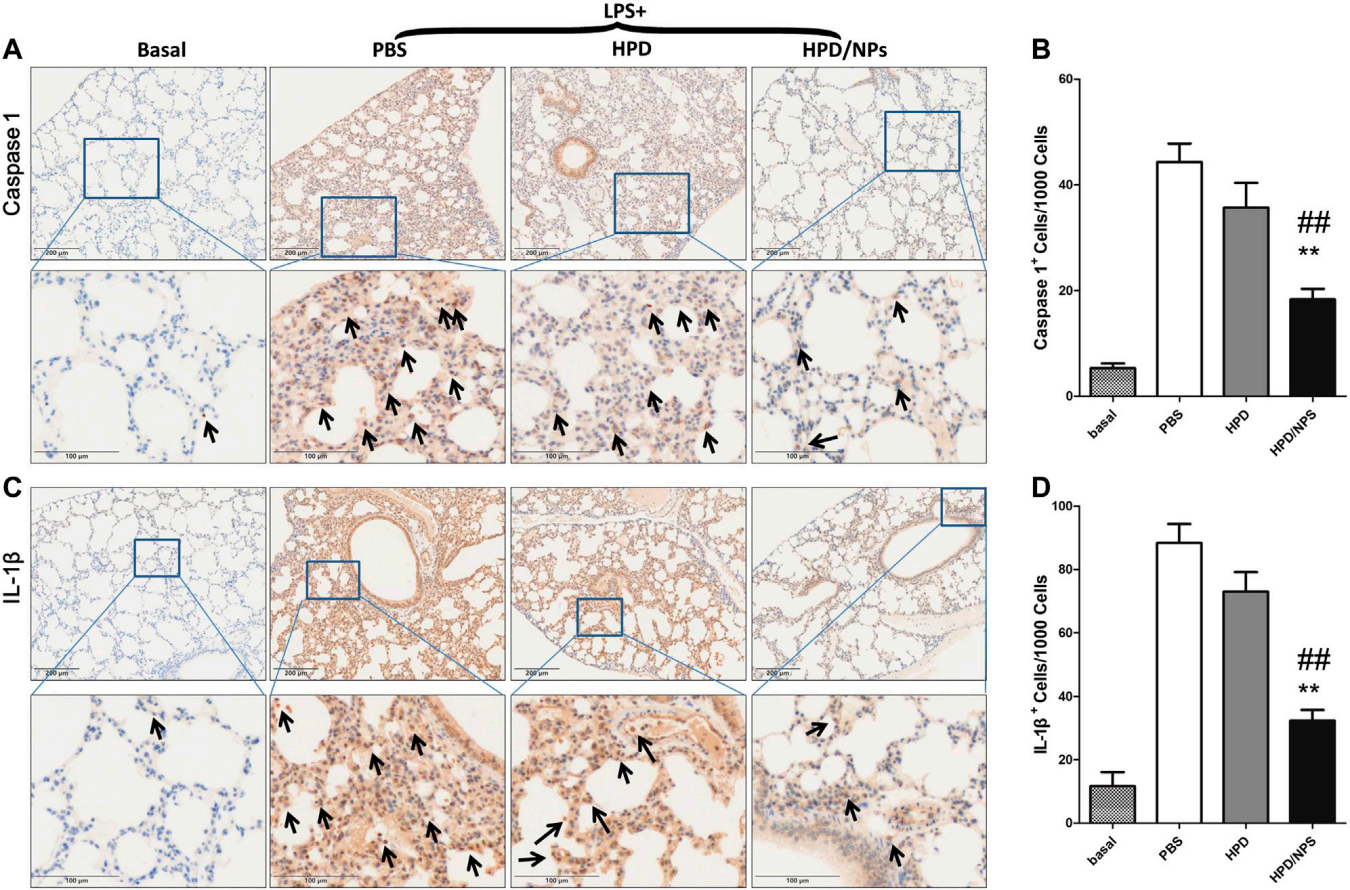
Impact of HPD and HPD/NPs on markers of pyroptosis in lungs of LPS-challenged mice. At 3 h post-LPS, PBS (vehicle), HPD, or HPD/NPs were nasally administered to mice. Lung tissues were collected at 24 h post-LPS challenge. **(A)** Representative images and **(B)** quantification of lung tissue cross-sections immune-stained for caspase 1. **(C)** Representative images and **(D)** quantification of lung tissue cross-sections immune-stained for IL-1β. Arrows indicate brown/positive staining. *N* = 4/group; **p < 0.01 vs. PBS, ^##^ *p* < 0.01 vs. HPD.

To investigate whether cell pyroptosis (Bergsbaken et al., 2009) was involved in the pathogenesis of ALI in LPS-treated mice and to explore the possible inhibition of LPS-induced pyroptosis by HPD/NPs, immunohistochemical staining was performed to detect the expression levels of IL-1β and caspase 1 in the lung of septic mice (Figure 6). The septic lung tissues in the PBS-treated group showed significantly increased expressions of IL-1β and caspase 1 compared with mice in the basal group. Excitingly, the expression of IL-1β and caspase 1 decreased significantly following treatment of LPS mice with HPD/NPs. The inhibitory effect of HPD on pyroptosis was once more less than that of HPD/NPs, again suggesting that these modified NPs can be used to improve drug delivery and treatment efficacy in inflammatory lung.

## Discussion

ALI following sepsis or infection with SARS-CoV, MERS-CoV, or SARS-CoV-2, represent major healthcare and financial problems worldwide (Su et al., 2016). Despite the threats to human survival and well-being, there are still no effective therapeutic drugs against ALI/ARDS. Additionally, human studies have shown that patients with severe COVID-19 also demonstrate CSS (He et al., 2020). Thus, the develop of novel drugs or treatment strategies against ALI/ARDS and/or CSS is of upmost importance. Wu et al. (Wu et al., 2020) employed computational methods to identify therapeutic targets for COVID-19 and discovered that HPD could be used as a potential anti- COVID-19 drug. Although this anti-inflammatory and anti-viral agent (Lin et al., 2005) can be easily extracted from *Isatis indigotica* roots and phenolic Chinese herbs, and was extensively used for the prevention of SARS in China, the poor water solubility and bioavailability of HPD limit its efficiency, especially when delivery intranasally.

Herein, we designed a HPD/NP delivery system to target inflammatory lung tissue and reduce CSS. To improve the adsorption rate of the HPD/NPs by nasal mucosa, chitosan was added to the surface of the PLGA NPs (Garg et al., 2019). The zeta potential of the HPD/NPs was +22 mV, suggestive of good adsorption of HPD/NPs into nasal mucosa. However, the impact of different types of NP on nasal mucosal absorption per se should be assessed in future studies.

*In vitro*, we identified 10 µg/ml as a dose of HPD/NPs that does not induce cytotoxicity in RAW264.7 cells, while 50 µg/ml or 100 µg/ml of both HPD and HPD/NPs did decrease cell viability. Thus, the dose of 10 µg/ml was selected to be assessed in the *in vitro* and *in vivo* experiments. While both HPD and HPD/NPs could significantly inhibit the production of inflammatory cytokines (NO and IL-6) *in vitro*, this inhibition was greater when cells were treated with HPD/NPs compared with HPD alone. *In vivo*, 10 mg/kg of HPD/NPs but not free HPD significantly suppressed CSS. These studies suggest that the anti-inflammatory impact of HPD is enhanced by delivery in chitosan NPs. This delivery system also alleviated the need for DMSO as a solubility agent *in vivo*, which could damage nasal mucosa when administrated nasally. Furthermore, excessive amounts of NO can promote cytokine and matrix metalloproteinase production, mitochondrial dysfunction, and cell apoptosis, which aggravates inflammation and tissue injury (Wu et al., 2018). The HPD/NPs significantly inhibited NO release in LPS-challenged mice, implying that nasal delivery of HPD/NPs can enhance the anti-CSS effects of HPD through reductions in multiple different cytokines and signaling molecules.

To determine the impact of HPD/NPs on CSS-associated ALI, we next investigated the effects of HPD/NPs on endothelial barrier function, which plays a vital role in lung inflammation and ALI We showed that the damaged integrity of endothelial monolayers following LPS treatment could be restored by HPD/NP treatment *in vitro*. Consistent with these findings, we also showed that nasal HPD/NPs significantly attenuated LPS-induced increases in ALI in mice, as shown by reductions in vascular permeability, CSS, infiltration of leukocytes, and presence of protein-rich liquid in pulmonary alveoli. Importantly, we showed that the NPs alone did not alter inflammatory cytokine levels or NO production in LPS-treated mice, suggesting that HPD/NPs but not the NPs alone were active pharmacological agents against CSS-associated ALI. Pyroptosis is involved in the development of inflammatory lung diseases such as ALI/ARDS, and we demonstrated that the HPD/NPs inhibited pulmonary cell pyroptosis markers in LPS-treated mice. These findings together support the possibility that HPD/NPs reduce CSS-associated ALI through decreases in cytokine release, vascular permeability, and cell pyroptosis, and have potential for the development of novel treatment strategies for sepsis and CSS in infectious diseases.

It is worth mentioning that the HPD dose that had protective effects on CSS-induced lung injury in our study is 5-fold lower than HPD dose in free form used to reduce smoke-induced lung inflammation in a previous study (Yu et al., 2019). Previous experimental studies have shown that treatment efficacy can be improved through delivery strategies that target inflammatory tissues (Zhang et al., 2019) (Gao et al., 2019). In this work, the anti-inflammatory effects of HPD on macrophages and lungs of LPS-challenged mice was improved by using the nasal NP-based drug delivery system. In summary, our data provide strong evidence that nasal NP-delivery of HPD protects against CSS-associated ALI and that the nasal NP delivery system could be used to enhance the efficacy of anti-inflammatory agents in the treatment of CSS and ALI/ARDS.

## Data Availability Statement

The raw data supporting the conclusions of this article will be made available by the authors, without undue reservation.

## Ethics Statement

The animal study was reviewed and approved by Guangdong Medical University.

## Author Contributions

HJ, ZZ, and XL proposed and supervised the project. HJ, QL, ZM, HZ, YW, XD, WZ, and JP performed the experiments. HJ wrote the paper. CE revised and polished the manuscript. XL provided the funding in this study. All authors have given approval to the final version of the manuscript.

## Funding

This work was supported by National Natural Science Foundation of China (No. 81971329, 81671399), Special Funds for the Cultivation of Guangdong College Students’Scientific, Technological Innovation (Climbing Program Special Funds, No. pdjh2019b0224), PhD early development program of Guangdong medical University (B2019012) and “Group-type” Special Supporting Project for Educational Talents in Universities (4SG19057G). CE received an American Heart Association Career Development Award (19CDA34500000).

## Conflict of Interest

The authors declare that the research was conducted in the absence of any commercial or financial relationships that could be construed as a potential conflict of interest.
